# The Impact of High-Flow Nasal Cannula on the Outcome of Immunocompromised Patients with Acute Respiratory Failure: A Systematic Review and Meta-Analysis

**DOI:** 10.3390/medicina55100693

**Published:** 2019-10-16

**Authors:** Li-Chin Cheng, Shen-Peng Chang, Jian-Jhong Wang, Sheng-Yen Hsiao, Chih-Cheng Lai, Chien-Ming Chao

**Affiliations:** 1 Divisin of Colorectal Surgery, Department of Surgery, Chi Mei Medical Center, Tainan 71004, Taiwan; leproxy@gmail.com; 2 Yijia Pharmacy, Tainan 70846, Taiwan; httremoon@ms.szmc.edu.tw; 3 Division of Nephrology, Department of Internal Medicine, Chi Mei Medical Center, Chiali 72263, Taiwan; win7a@yahoo.com.tw; 4 Department of Internal Medicine, Chi Mei Medical Center, Liouying 73657, Taiwan; seedvirt@hotmail.com; 5 Department of Internal Medicine, Kaohsiung Veterans General Hospital, Tainan Branch, Tainan 71051, Taiwan; dtmed141@gmail.com; 6 Department of Intensive Care Medicine, Chi Mei Medical Center, Liouying 73657, Taiwan

**Keywords:** high-flow nasal cannula, immunocompromised, non-invasive ventilation, intubation, mortality, acute respiratory failure

## Abstract

*Background and objectives*: High-flow nasal cannula (HFNC) can be used as a respiratory support strategy for patients with acute respiratory failure (ARF). However, no clear evidence exists to support or oppose HFNC use in immunocompromised patients. Thus, this meta-analysis aims to assess the effects of HFNC, compared to conventional oxygen therapy (COT) and noninvasive ventilation (NIV), on the outcomes in immunocompromised patients with ARF. The Pubmed, Embase and Cochrane databases were searched up to November 2018. *Materials and Methods*: Only clinical studies comparing the effect of HFNC with COT or NIV for immunocompromised patients with ARF were included. The outcome included the rate of intubation, mortality and length of stay (LOS). *Results*: A total of eight studies involving 1433 immunocompromised patients with ARF were enrolled. The pooled analysis showed that HFNC was significantly associated with a reduced intubation rate (risk ratio (RR), 0.83; 95% confidence interval (CI), 0.74–0.94, *I*^2^ = 0%). Among subgroup analysis, HFNC was associated with a lower intubation rate than COT (RR, 0.86; 95% CI, 0.75–0.95, *I*^2^ = 0%) and NIV (RR, 0.59; 95% CI, 0.40–0.86, *I*^2^ = 0%), respectively. However, there was no significant difference between HFNC and control groups in terms of 28-day mortality (RR, 0.78; 95% CI, 0.58–1.04, *I*^2^ = 48%), and intensive care unit (ICU) mortality (RR, 0.87; 95% CI, 0.73–1.05, *I*^2^ = 57%). The ICU and hospital LOS were similar between HFNC and control groups (ICU LOS: mean difference, 0.49 days; 95% CI, −0.25–1.23, *I*^2^ = 69%; hospital LOS: mean difference, −0.12 days; 95% CI, −1.86–1.61, *I*^2^ = 64%). *Conclusions*: Use of HFNC may decrease the intubation rate in immunocompromised patients with ARF compared with the control group, including COT and NIV. However, HFNC could not provide additional survival benefit or shorten the LOS. Further large, randomized controlled trials are needed to confirm these findings.

## 1. Introduction 

With the aggressive surveillance and the improvement of medical care, the incidence of cancer, as well as the survival of cancer patients, are both increasing steadily [1,2,3]. The populations of immunocompromised patients, including active cancer, organ transplant, the use of immunosuppressive agents and chemotherapy, and human immunodeficiency virus (HIV) infections, are growing [4,5,6]. However, immunocompromised patients also carry higher risks of many life-threatening complications than do immunocompetent patients. Infection-related acute respiratory failure (ARF) requiring invasive mechanical ventilation (IMV) is the most common cause of the immunocompromised patients needing intensive care unit (ICU) admissions [7,8,9,10]. Moreover, immunocompromised patients requiring IMV for ARF in the ICU usually have high morbidity and mortality [9,10,11]. Instead of IMV, non-invasive respiratory support is an established alternative treatment for ARF among immunocompromised patients. However, a multicenter, randomized clinical study [12] showed that non-invasive ventilation (NIV) did not provide an additional survival benefit among immunocompromised patients compared with standard oxygen therapy. Recently, the development of high-flow nasal-cannula (HFNC) gives us another technique of non-invasive respiratory supports for ARF. HFNC has several advantages, including high oxygen flows with a high fraction of inspired oxygen, the generation of flow-dependent positive end-expiratory pulmonary pressure, and enhanced wash out of nasopharyngeal dead space, but without compromising blood flow to skin areas susceptible to pressure sores [13,14,15]. Two recent meta-analyses [16,17] showed that HFNC was associated with a lower rate of intubation than conventional oxygen therapy (COT) in adult patients with acute hypoxemic respiratory failure. In contrast, several meta-analyses [18,19] demonstrated that HFNC was not associated with a significant difference in mortality compared to COT in patients with acute hypoxemic respiratory failure. For immunocompromised patients, prolonged endotracheal intubation could be associated with a high rate of infection and poor outcome. If HFNC can help lower the intubation rate among immunocompromised patients, their outcome may be improved. However, only one meta-analysis [20] of seven studies found that HFNC was significantly associated with a reduction in short-term mortality and intubation rate. In this meta-analysis, only 667 ARF patients and one randomized controlled trial (RCT) were included [20]. In 2018, one large multicenter RCT [21] involving 776 patients showed that HFNC did not significantly decrease the day-28 mortality and intubation rate compared with COT. Therefore, we conduct this updated meta-analysis, incorporating these studies with conflicting results to increase the evidence level and power of analysis, and aim to find out the answer about the clinical efficacy of HFNC in immunocompromised patients with ARF.

## 2. Materials and Methods

### Study Search and Selection

All clinical studies were identified by a systematic review of the literature in the PubMed, Embase, and Cochrane databases until 10 November 2018, using the following Mesh terms—“high-flow nasal cannula”, “nasal high flow”, “humidified high-flow nasal cannula”, “respiratory failure”, “acute hypoxemic respiratory failure” and “acute respiratory failure”. Only randomized controlled trial (RCT) or observational studies that compared the clinical efficacy of High-flow nasal cannula (HFNC) and conventional oxygen therapy (COT) or noninvasive ventilation (NIV), for immunocompromised adult patients with acute respiratory failure (ARF), were included. Immunocompromised status was defined as a chronic use of steroids, the use of other immunosuppressant or chemotherapeutic agents, solid organ transplantation, solid and hematologic malignancy, human immunodeficiency virus (HIV) infection, or primary immune deficiency. In addition, we searched all references in the relevant articles and reviews for additional eligible studies. We excluded case reports or case series, single arm studies, studies enrolling pediatric patients on neonate, and conference abstracts. Two reviewers (Chang and Wang) searched and examined publications independently to avoid bias. When they had any disagreement, another author (Lai) resolved the issue in time. 

The following data included authors, year of publication, study design and duration, sites of study, the demographic characteristics of the study population, immunocompromised conditions, disease severity, indication of oxygen therapy for ARF, and the outcomes. Neither ethics board approval nor patient consent was required, due to the nature of a systematic review. The Cochrane Risk for Bias Assessment tool [22] and modified Newcastle-Ottawa scale [23] were used to assess the risk of bias for RCTs, and the cohort study, respectively.

The primary outcome was the rate of intubation. Secondary outcomes included all-cause mortality, including 28-day, intensive care unit (ICU) or hospital mortality and length of stay (LOS) in the ICU and hospital. The statistical analysis was conducted using the software Review Manager, Version 5.3. The degree of heterogeneity was evaluated with the *Q* statistic generated from the χ^2^ test. The proportion of statistical heterogeneity was assessed by the *I*^2^ measure. Heterogeneity was considered as significant when its *p*-value was less than 0.10, or the *I*^2^ more than 50%. The fixed-effect model and the random-effects model were applied when the data was homogenous, and heterogeneous, respectively. For dichotomous outcomes, we estimated the summary risk ratio and 95% confidence interval (CI); for continuous data, we estimated summary mean difference (MD) and 95% CIs. The mean and standard deviations were estimated from the median and interquartile ranges according to a previous study [24]. A *p*-value <0.05 was set as the threshold of statistical significance. Sensitivity analyses were conducted by excluding or subgrouping studies to reduce the potential confounding effects.

## 3. Results

### 3.1. Study Selection and Characteristics

**Figure 1:**
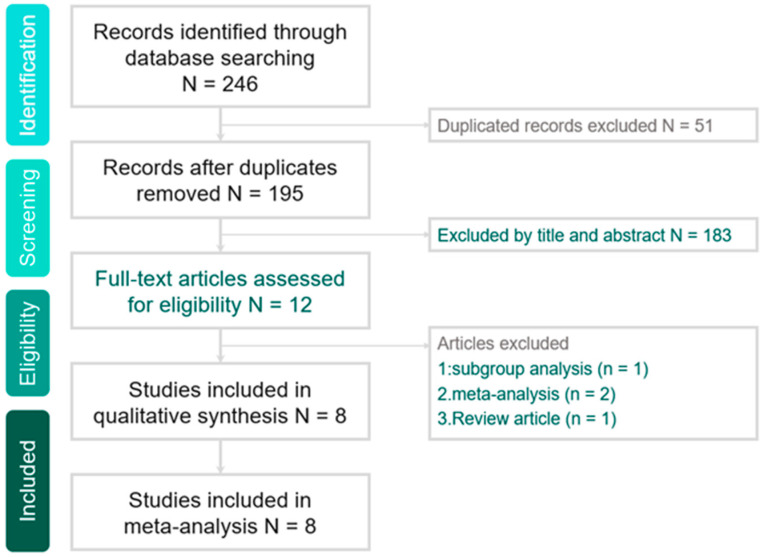
Study selection.

The search program yielded 246 references, including 53 from Pubmed, 169 from Embase and 24 from the Cochrane database. Then, 195 articles were screened after excluding 51 duplicated articles. Finally, a total of eight studies [21,25,26,27,28,29,30,31] fulfilling the inclusion criteria were included in this meta-analysis (Figure 1). All were studies designed to compare the clinical efficacy of HFNC and COT or NIV for immunocompromised patients with ARF (Table 1). The risk of bias is shown in the Appendix A ( Table A1 and Table A2). The risk of blinding was high in all RCTs. Four studies were retrospective studies [25,26,29,31], two studies [27,30] were post-hoc analyses of RCT, and two studies [21,28] were RCTs. In the Frat et al. study [30], we only extracted 26 and 30 patients who received HFNC and COT, respectively, and we did not enroll the other 26 patients using HFNC plus NIV. Four studies [25,26,29,31] were conducted in a single center, and the other four were multicenter studies [21,27,28,30]. During the initial enrollment, HFNC and comparator were applied for 727 and 716 patients, respectively. Except for the fact that one study [29] was conducted in Asia, the other seven studies [21,25,26,27,28,30,31] were performed in Europe. Five studies [21,26,27,28,30] used COT as comparator. Two studies [29,31] assessed HFNC versus NIV alone. In the Mokart et al. study [25], they evaluated HFNC plus NIV versus NIV alone (Table 2).

### 3.2. Primary Outcomes

**Figure 2:**
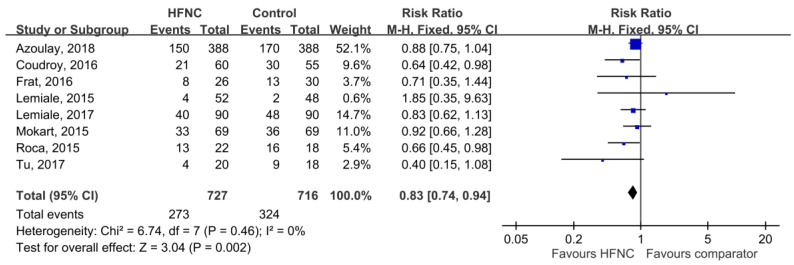
Association of high-flow nasal cannula (HFNC) with rate of intubation.

Among all of the eight enrolled trials, the intubation rate was 37.6% (273/727) and 45.3% (324/716) in the group of patients who were assigned to HFNC and controls, respectively. The pooled analysis showed that HFNC was significantly associated with a reduced intubation rate (RR, 0.83; 95% CI, 0.74–0.94, *I*^2^ = 0%, Figure 2). Sensitivity analysis after deleting an individual study each time to reflect the influence of the single dataset on the pooled RR showed similar findings. Among subgroup analysis, HFNC was associated with lower intubation rate than COT (RR, 0.86; 95% CI, 0.75–0.95, *I*^2^ = 0%) and NIV (RR, 0.59; 95% CI, 0.40–0.86, *I*^2^ = 0%), respectively (Table 3). According to a different study design, we found HFNC significantly reduced the intubation rate in retrospective studies (RR, 0.73; 95% CI, 0.58–0.90, *I*^2^ = 21%). In contrast, no difference was found in two pooled RCTs (RR, 0.89; 95% CI, 0.76–1.06, *I*^2^ = 0%) and two post-hoc analyses of RCTs (RR, 0.81; 95% CI, 0.61–1.07, *I*^2^ = 0%) (Table 3). In the pooled analysis of four studies conducted in the single center, HFNC was associated with a lower intubation rate than the control group (RR, 0.73; 95% CI, 0.58–0.90, *I*^2^ = 21%), but no difference was found in four multicenter studies (RR, 0.87; 95% CI, 0.76–1.01, *I*^2^ = 0%).

### 3.3. Secondary Outcomes

**Figure 3:**
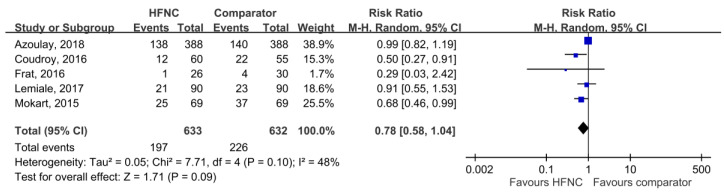
Association of high-flow nasal cannula (HFNC) with 28-day mortality.

**Figure 4:**
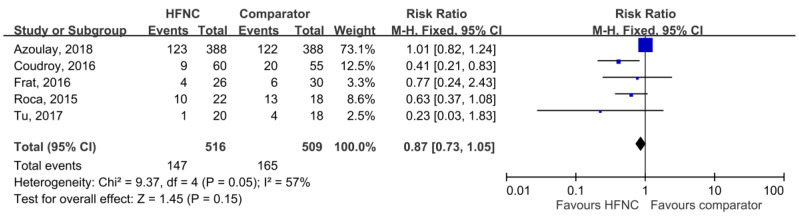
Association of high-flow nasal cannula (HFNC) with intensive care unit (ICU) mortality.

**Figure 5:**
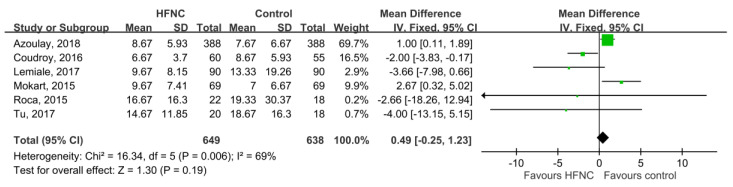
Association of high-flow nasal cannula (HFNC) with length of ICU stay.

**Figure 6:**
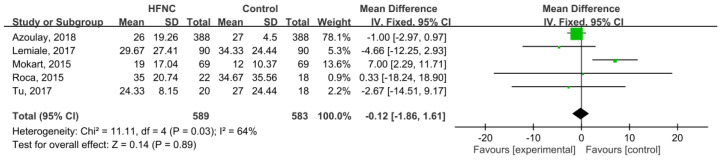
Association of high-flow nasal cannula (HFNC) with length of hospital stay.

28-day mortality was available in five studies [21,25,27,30,31], and the pooled results found that there was no significant difference between HFNC and the control groups (RR, 0.78; 95% CI, 0.58–1.04, *I*^2^ = 48%, Figure 3). Five studies [21,26,29,30,31] reported ICU mortality, and no significant difference was found between HFNC and the control group (RR, 0.87; 95% CI, 0.73–1.05, *I*^2^ = 57%, Figure 4). Six studies [21,25,26,27,29,31] reported ICU LOS, and five studies [21,25,26,27,29] showed hospital LOS. We found ICU and hospital LOS were similar between HFNC and control groups (ICU LOS: Mean difference, 0.49 days; 95% CI, −0.25 to 1.23, *I*^2^ = 69%; hospital LOS: Mean difference, −0.12 days; 95% CI, −1.86 to 1.61, *I*^2^ = 64%) (Figure 5 and Figure 6).

## 4. Discussion

This meta-analysis enrolled a total of eight studies involving immunocompromised patients with ARF and provided several significant findings. Most importantly, the use of HFNC can help reduce the intubation rate in immunocompromised patients with ARF. The effect remained significant in the subgroup analysis while compared with COT and NIV. The similar findings have been reported in previous studies [32] in other clinical conditions. For post-extubation patients, HFNC treatment significantly decreased the reintubation rate (OR 0.46; 95%CI 0.33–0.63; *p* <  0.00001; *I*^2^ = 30%) and extubation failure (OR 0.43; 95%CI 0.25–0.73; *p* = 0.002; *I*^2^ = 66%) when compared with COT in a meta-analysis [32] of 18 RCTs. Another meta-analysis of six RCTs demonstrated that HFNC therapy can decrease the intubation rate (RR, 0.60; 95% CI, 0.28–0.94, *I*^2^ = 49) when ARF patients were treated with HFNC therapy, compared with COT [16]. Even for immunocompromised patients with ARF, the meta-analysis of Huang et al. [20] showed the similar findings that HFNC was significantly associated with a reduction in the intubation rate (RR, 0.76; 95% CI, 0.64–0.90). Overall, our meta-analysis remained consistent with previous suggestions that HFNC may help prevent intubation among immunocompromised patients with ARF compared with COT and NIV.

However, the significant effect of HFNC in reducing the intubation rate was only evident in the pooled analysis of four retrospective studies or single center investigations. In contrast, no significant difference was noted in the pooled analysis of two RCTs, two post-hoc analysis of RCTs, or four multicenter studies. This may be explained by the different study design. In the single center, the treatment protocol may be more consistent than in the multicenter RCT trial. In addition, none of the studies were double-blinded clinical trials, due to the nature of this kind of study. The lack of blinding may affect the treatment and the following assessment of the outcomes. Although our finding seems robust due to the low heterogeneity in the analysis of primary outcome, we still needed more solid data from large scale RCTs to confirm our findings.

Despite finding a positive impact of HFNC on reducing intubation rates among immunocompromised patients with ARF, there was no significant difference between HFNC and control groups regarding secondary outcomes, including 28-day mortality or ICU mortality, and length of ICU or hospital stays. Although the level of evidence regarding our findings of secondary outcomes was low due to the relative high heterogeneity and limited RCTs, these findings can be explained by that the causes of ARF, and the characteristics of study populations are protean, and the treatment of ARF is complicated. As a lone intervention, HFNC cannot improve overall outcomes including mortality and LOS. In addition, HFNC might not reduce mortality (despite reducing intubation rates) because of potential harm caused in the patients that were eventually intubated. In these patients, intubation may have been delayed, and outcomes made comparatively worse.

Although this meta-analysis is updated, and includes double the number of patients than the previous meta-analysis, there were several limitations within this study. First, there was relatively high heterogeneity with an *I*^2^ value of more than 50% in the secondary outcome analysis. These heterogeneities could be caused by some significant variations in study design, and the clinical characteristics of enrolled patients. Second, most of the participating ICUs were located in Europe, especially in France, so the generalizability may be limited.

## 5. Conclusions

This meta-analysis suggested that the use of HFNC may decrease the intubation rate in immunocompromised patients with ARF compared with the control group, including COT and NIV. However, HFNC could not provide additional survival benefit or shorten the LOS in the ICU or hospital. Further large, high-quality, randomized, multi-center trials are needed to confirm the effects of HFNC among immunocompromised patients with ARF.

## Figures and Tables

**Table 1:** Characteristics of enrolled studies.

Study, Year	Study Design	Study Period	Study Site	Immunocompromised Condition	Inclusion Criteria
Tu, 2017	Retrospective	2011–2015	Single tertiary mixed ICU in China	Renal transplant	RR > 25/min, PaO_2_/FiO_2_ ≤ 200 mm Hg, PaCO_2_ ≤ 45 mm Hg
Coudroy, 2016	Retrospective	2007–2014	Single tertiary medical ICU in France	Hematologic or solid cancer, stem or solid organ transplantation, steroid, cytotoxic drug, AIDS	RR > 25/min, sign of respiratory distress, PaO_2_/FiO_2_ ≤ 300 mm Hg
Frat, 2016	Post-hoc analysis of RCT	2011–2013	23 ICUs in France and Belgium	Solid or hematological cancer, AIDS, immunosuppressive drug or steroid	RR > 25/min, PaO_2_/FiO_2_ ≤ 300 mm Hg, PaCO_2_ ≤ 45 mm Hg
Lemiale, 2017	Post-hoc analysis of RCT	2013–2015	28 ICUs in France and Belgium	Hematologic malignancy or solid tumor, solid organ transplant, long-term or high-dose steroid, immunosuppressive drug	PaO_2_ < 60 mm Hg on room air or tachypnea> 30/min or symptoms of respiratory distress
Lemiale, 2015	Open, parallel-group RCT	2012–2014	4 ICUs in France	Solid or hematological cancer, solid organ transplant, long-term or high-dose steroid, immunosuppressive drug, HIV infection	O_2_ delivery >6 L/min to maintain SpO_2_ > 95% or symptoms of respiratory distress
Mokart, 2015	Retrospective	2009–2014	Single center in France	Cancer	Severe acute respiratory failure (O_2_ delivery >9 L/min)
Roca, 2015	Retrospective	2007–2011	Single center in Spain	Lung transplant	Inability to maintain SpO_2_ > 95% and an RR ≥ 25/min
Azoulay, 2018	RCT	2016–2017	32 ICUs in France	Hematologic malignancy or solid tumor, solid organ transplant, long-term or high-dose steroid, immunosuppressive drug, primary immune deficiency	PaO_2_ < 60 mm Hg or SpO_2_ < 90% on room air or tachypnea >30/min or symptoms of respiratory distress, need for oxygen ≥6 L/min

RCT, randomized controlled trial; ICU, intensive care unit; RR, respiratory rate; IMV, invasive mechanical ventilation; NIV, non-invasive ventilation; AIDS, acquired immune deficiency syndrome; HIV human immunodeficiency virus.

**Table 2:** Characteristics of study population.

Study, Year	No. of Patients		Mean Age		Percentage of Common ARF Etiology		Severity	
	HFNC	Control Group	HFNC	Control Group	HFNC	Control Group	HFNC	Control Group
Tu, 2017	20	18 (NIV)	47	47	NA	NA	SAPS II 37 (4)	SAPS II 35 (6)
Coudroy, 2016	60	55 (NIV)	58	62	Infection (44%), cardiogenic edema (9%)	Infection (52%), cardiogenic edema (8%)	SAPS II 42 (11) SOFA 3 (1–6)	SAPS II 46 (13) SOFA 4 (1–6)
Frat, 2016	26	30 (COT)	62	63	Pneumonia (66%), cancer (12%), others (23%)	Pneumonia (94%), cancer (3%), others (3%)	SAPS II 29 (11)	SAPS II 30 (17)
Lemiale, 2017	90	90 (COT)	64	63	Infection (73.3%), cardiogenic edema (2.2%) other (24.4%)	Infection (68.8%), cardiogenic edema (2.2) other (28.8%)	SOFA 4 (2–6)	SOFA 3 (2–6)
Lemiale, 2015	52	48 (COT)	50	49	Sepsis (48.1%), PJP (9.6%), cardiogenic edema (9.6%)	Sepsis (52%), PJP (4.1%), cardiogenic edema (4.1%)	SOFA 3.5 (2–6) SAPS II 42 (29.5–52)	SOFA 3 (2–5) SAPS II 37.5 (31–47)
Mokart, 2015	69 (HFNC + NIV)	59 (NIV + COT)	56	59	Pulmonary sepsis (65%), cancer (19%), others (26%)	Pulmonary sepsis (65%), cancer (9%), others (43%)	SOFA 6 (4–8) SPAS II 47 (37–55)	SOFA 6 (4–9) SAPS 48 (3859)
Roca, 2015	22	18 (COT)	56	53.5	Infection (91.0%), rejection (4.5%)	Infection (72.2%), rejection (5.6%)	SOFA 4 (4–6)	SOFA 4 (4–6)
Azoulay, 2018	388	388 (COT)	64	63	NA	NA	SAPS II 36 (28–46); SOFA 6 (4–8)	SAPS II 37 (28–48); SOFA 6 (4–8)

HFNC, high-flow nasal cannula; COT, conventional oxygen therapy; NIV, non-invasive ventilation; NA, not available; ARF, acute respiratory failure; PJP, *Pneumocystis jiroveci* pneumonia.

**Table 3:** Subgroup analysis.

Subgroup	No of Study	No of Patients		Random-Effect Model		*I*^2^ (%)	Test of Heterogeneity P
		HFNC	Control	Risk Ratio	95% CI		
Comparator							
HFNC vs. COT	5	578	574	0.86	0.75–0.95	0	0.57
HFNC vs. NIV	2	80	73	0.59	0.40–0.86	0	0.39
HFNC + NIV vs. COT + NIV	1	69	69	0.92	0.66–1.28	NA	NA
Study design							
RCT	2	440	436	0.89	0.76–1.06	0	0.38
Retrospective study	4	171	160	0.73	0.58–0.90	21	0.28
Post-hoc analysis	2	116	120	0.81	0.61–1.07	0	0.68
Study site							
Single center	4	171	160	0.73	0.58–0.90	21	0.28
Multicenter	4	556	556	0.87	0.76–1.01	0	0.88

HFNC, high-flow nasal cannula; COT, conventional oxygen therapy; NIV, non-invasive ventilation; RCT, randomized controlled trial.

## Appendix A

**Table A1:** Risk of bias of randomized controlled trials (RCTs).

Reference	Random Sequence Generation	Allocation Concealment	Blinding of Participants and Personnel	Blinding of Outcome Assessment	Incomplete Outcome Data	Selective Reporting
Frat, 2016	Low	High	High	High	Low	Low
Lemiale, 2017	Low	High	High	High	Low	Low
Lemiale, 2015	Low	High	High	High	Low	Low
Azoulay, 2018	Low	Low	High	High	Low	Low

**Table A2:** Newcastle-Ottawa scale for observational studies.

Reference	Representative of Exposed Cohort	Selection of Non-Exposed Cohort	Ascertainment of Exposure	Demonstration that Outcome Was Not Present at Start of Study	Comparability of Cohorts Based on Design and Analysis	Assessment of Outcome	Timing of Follow-Up	Adequate Follow-Up	Score
Mokart, 2015	V	V	V	V	V	V	V	V	8
Roca, 2015	V	V	V	V	V	V	V	V	8
Coudroy, 2016	V	V	V	V	V	V	V	V	8
Tu, 2017	V	V	V	V	V	V	V	V	8
